# Machine learning-based strategies for improving healthcare data quality: an evaluation of accuracy, completeness, and reusability

**DOI:** 10.3389/frai.2025.1621514

**Published:** 2025-07-21

**Authors:** Agate Jarmakovica

**Affiliations:** Faculty of Computer Science, Information Technology and Energy, Riga Technical University, Riga, Latvia

**Keywords:** data quality, machine learning, accuracy, completeness, reusability, healthcare data analysis

## Abstract

Healthcare data quality is a critical factor in clinical decision-making, diagnostic accuracy, and the overall efficacy of healthcare systems. This study addresses key challenges such as missing values and anomalies in healthcare datasets, which can result in misdiagnoses and inefficient resource use. The objective is to develop and evaluate a machine learning-based strategy to improve healthcare data quality, with a focus on three core dimensions: accuracy, completeness, and reusability. A publicly available diabetes dataset comprising 768 records and 9 variables was used. The methodology involved a comprehensive data preprocessing workflow, including data acquisition, cleaning, and exploratory analysis using established Python tools. Missing values were addressed using K-nearest neighbors imputation, while anomaly detection was performed using ensemble techniques. Principal Component Analysis (PCA) and correlation analysis were applied to identify key predictors of diabetes, such as Glucose, BMI, and Age. The results showed significant improvements in data completeness (from 90.57% to nearly 100%), better accuracy by mitigating anomalies, and enhanced reusability for downstream machine learning tasks. In predictive modeling, Random Forest outperformed LightGBM, achieving an accuracy of 75.3% and an AUC of 0.83. The process was fully documented, and reproducibility tools were integrated to ensure the methodology could be replicated and extended. These findings demonstrate the potential of machine learning to support robust data quality improvement frameworks in healthcare, ultimately contributing to better clinical outcomes and predictive capabilities.

## 1 Introduction

Healthcare data quality is a critical determinant of clinical decision-making, diagnostic accuracy, and overall patient outcomes. According to ISO 9000 quality guidelines, quality is defined as the extent to which a product or service meets customer needs by embodying all the necessary characteristics to achieve its intended purpose (ISO, 2015). In healthcare, data are treated as a “product” that must be tailored to specific user requirements, such as supporting clinical decisions or developing machine learning models. This dynamic and context-dependent nature of data quality is especially significant in healthcare, where the availability of high-quality data directly influences clinical prognoses and treatment outcomes.

The importance of data quality in healthcare is underscored by the potential consequences of poor-quality data, which may lead to misdiagnoses, treatment errors, and inefficient resource utilization. Prior studies have highlighted that data quality is intrinsically linked to dimensions such as accuracy, completeness, consistency, and timeliness (Liu et al., 2023). For instance, Moses et al. (2021) describe data quality as the “health” of data across its lifecycle—from acquisition to analysis—emphasizing its impact on operational efficiency and decision-making.

Moreover, the literature reveals that effective data quality improvement requires both technical and organizational approaches. Brownlee (2020) outlines a multi-step data cleaning process that includes missing value imputation, removal of duplicate records, noise reduction, and normalization. Industry sources support this view, highlighting that effective data quality strategies also require proactive monitoring, metadata-driven automation, and stakeholder alignment across data pipelines (Acceldata, 2024). Techniques such as the k-nearest neighbors (KNN) imputation (Thomas and Rajabi, 2021) and anomaly detection methods like Isolation Forest and Local Outlier Factor (LOF) (Liu et al., 2008) have been shown to effectively address common issues such as missing values and outliers, which are particularly prevalent in healthcare datasets.

The open science movement further emphasizes the necessity for broad access to research methods and datasets to foster collective innovation, reproducibility, and transparency in scientific research (Open Science Guidebook, 2024). However, this approach must be balanced with stringent data protection requirements to safeguard patient confidentiality and data security.

Given the critical role of data quality in ensuring reliable clinical outcomes and the proven impact of quality issues on machine learning model performance (Gong et al., 2023; Wei et al., 2025; Miller et al., 2024), this study aims to develop and evaluate a machine learning-based strategy to enhance healthcare data quality. The strategy focuses on three key dimensions: accuracy, completeness, and reusability. Specifically, the study seeks to:

1. Assess the quality of healthcare datasets based on their accuracy, completeness, and fitness for purpose;
2. Apply state-of-the-art imputation and anomaly detection methods to mitigate data quality issues; and
3. Validate the effectiveness of these methods through comparative analysis of predictive models.

By integrating advanced machine learning techniques with rigorous data preprocessing, this research contributes to the growing body of work dedicated to improving healthcare data quality—a critical prerequisite for reliable clinical decision-making and effective healthcare delivery.

## 2 Literature review

### 2.1 Data quality and its dimensions

Data quality is a fundamental element in any organizational process as it directly influences data usability, reliability, and the value derived during decision-making. In healthcare, poor data quality can result in severe consequences such as misdiagnosis, treatment errors, and increased operational costs, thereby compromising patient safety (Southekal, 2017). Early definitions of quality, including Crosby’s concept of “fit for purpose” (Crosby, 1979) and Redman’s focus on data suitability (Redman, 2016), have evolved into more nuanced frameworks. Moses et al. (2021) describe data quality as the “health” of data throughout its lifecycle—from acquisition to analysis—highlighting its critical impact on both clinical outcomes and operational efficiency. Schmidt et al. (2021) further categorize data quality into key dimensions—integrity, completeness, consistency, and accuracy—which ensure that data are structurally sound, fully populated, statistically precise, and contextually relevant. These dimensions are especially important in healthcare, where they underpin reliable clinical decisions and effective ML model performance (Wei et al., 2025). Recent developments by Miller et al. (2024) extend this perspective by proposing an expanded framework that incorporates modern challenges such as reproducibility, transparency, and ethical responsibility. These additional dimensions are essential in AI-driven healthcare environments, where accountability and explainability are as critical as technical correctness.

### 2.2 Data quality improvement

Improving data quality requires both technical and organizational interventions. Technically, processes such as deduplication, normalization, and adherence to data standards are essential. Brownlee (2020) outlines a multi-step data cleaning process that includes missing value imputation, duplicate removal, noise reduction, and data format alignment. Imputation methods—such as the k-nearest neighbors (KNN) algorithm—are particularly effective in healthcare settings where missing values are common (Thomas and Rajabi, 2021). Additionally, anomaly detection techniques like Isolation Forest and LOF are critical for identifying and correcting outliers, thereby enhancing overall data accuracy (Liu et al., 2008; Rakhi Gupta and Lamba, 2024). Furthermore, modern approaches to data quality emphasize the need to expand beyond traditional dimensions such as accuracy and completeness. Miller et al. (2024) propose a revised framework that includes traceability, reproducibility, and governability—factors that are increasingly critical in data-intensive environments such as healthcare and machine learning. These new dimensions ensure that data can be tracked across its lifecycle, validated through repeatable processes, and managed transparently within organizational systems, thus strengthening both operational and analytical reliability.

### 2.3 Data quality strategies in healthcare

Effective data quality strategies in healthcare are vital for clinical decision-making, diagnostic precision, and system efficiency. Loshin (2010) argues that such strategies must define explicit quality dimensions—namely accuracy, completeness, and consistency—and be embedded within a robust data management framework. Recent studies advocate for the incorporation of artificial intelligence and ML techniques to automate quality improvement. For example, Azimi and Pahl (2024) highlight the potential of ML-driven anomaly detection and correction, while Stanley and Schwartz (2024) stress the need for flexible, real-time monitoring systems that can adapt to evolving data landscapes. Industry sources similarly emphasize the importance of proactive, metadata-driven quality monitoring and alignment across stakeholders to ensure end-to-end data reliability (Acceldata, 2024). The present study’s objective—to develop a machine learning-based strategy focusing on accuracy, completeness, and reusability—aligns with these approaches, emphasizing that a balanced improvement across these dimensions is essential for successful data-driven healthcare solutions (Ehrlinger and Wöß, 2022; Moses et al., 2021). Data management is one of the most prominent challenges which complicates deep learning in industrial deployment. Although the deep learning technology has achieved very promising results, there is still a significant need for further research in the field of data management to build high-quality datasets and streams that can be used for building production-ready deep learning systems (Munappy et al., 2022).

### 2.4 Machine learning approaches in data analysis

Machine learning has emerged as a transformative tool for analyzing large and complex datasets. Sarker (2021) notes that ML techniques enable the extraction of hidden patterns, trend forecasting, and informed decision-making through data-driven insights. In healthcare, ML methods are particularly valuable for standardizing disparate data sources, addressing missing values, and detecting anomalies—factors that directly impact the performance of predictive models (Wei et al., 2025).

### 2.5 Supervised and unsupervised learning methods

**Figure 1:**
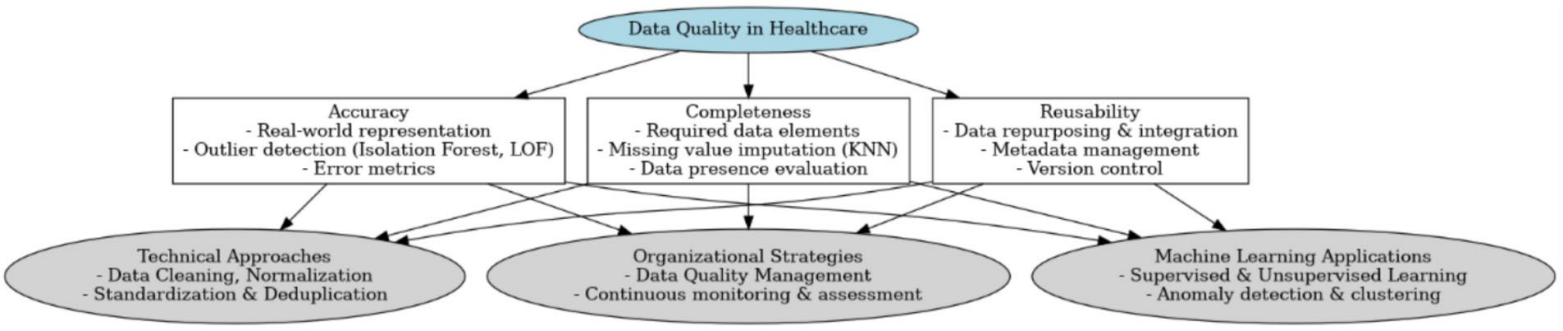
Conceptual framework for healthcare data quality.

ML techniques can be broadly classified into supervised and unsupervised learning methods. Supervised learning, which involves training models on pre-labeled data, is widely used for both classification and regression tasks (Müller and Guido, 2016). In contrast, unsupervised learning identifies inherent data structures, clusters, or outliers without pre-assigned labels, thereby enabling automated quality monitoring and improvement (Stanley and Schwartz, 2024; Usama et al., 2019). Such methods are particularly useful in validating the integrity and consistency of healthcare data. Figure 1 presents a visual summary of the key data quality dimensions—accuracy, completeness, and reusability—and illustrates the integration of technical methods (such as KNN imputation, Isolation Forest, and LOF with organizational strategies) and ML approaches to enhance overall data quality.

Based on the analyzed literature, the presented conceptual framework emphasizes that healthcare data quality hinges on three core dimensions—accuracy, completeness, and reusability—while integrating both technical and organizational approaches to ensure consistent, reliable, and adaptable data. Accuracy underscores the need for real-world representation and outlier detection (e.g., Isolation Forest, LOF), completeness focuses on mitigating missing values through imputation techniques such as KNN, and reusability stresses metadata management and version control to facilitate data repurposing. (Southekal, 2023). This is in line with insights from software engineering, where reuse mechanisms and traceability are identified as key drivers of transformation quality (Höppner and Tichy, 2024). This structure is supported by data cleaning, normalization, and deduplication (Brownlee, 2020), continuous monitoring and assessment within an organizational strategy (Loshin, 2010), and the incorporation of machine learning methods—both supervised and unsupervised—to automate anomaly detection and clustering (Müller and Guido, 2016; Stanley and Schwartz, 2024). By aligning these dimensions with advanced computational tools and robust management frameworks, the framework aims to enhance data reliability, thereby contributing to more accurate predictive modeling, improved clinical decision-making, and overall healthcare innovation (Zhao et al., 2024).

The literature reviewed provides a solid theoretical foundation for the current study, which aims to develop a machine learning-based strategy to enhance healthcare data quality. The reviewed sources emphasize that improvements in completeness (through imputation), accuracy (via anomaly detection), and reusability (through proper documentation) are interdependent and critical for reliable predictive analytics (Ehrlinger and Wöß, 2022; Moses et al., 2021).

## 3 Methods

The primary objective of this study is to develop and evaluate a machine learning-based strategy to improve healthcare data quality. The specific research aims are to (1) assess the current quality of healthcare datasets using defined metrics, (2) apply state-of-the-art imputation and anomaly detection methods to mitigate data quality issues, and (3) validate the effectiveness of these methods through a comparative analysis of predictive models. This aligns with practical guidance on implementing real-world data quality strategies, which emphasize measurable standards, scalable techniques, and integration into data workflows (Hawker, 2023). This research endeavors to contribute a systematic, reproducible approach to enhancing data integrity, ultimately supporting more accurate clinical analytics and informed healthcare decisions.

### 3.1 Data extraction, cleaning, and preparation

The dataset used in this study is publicly available on GitHub under the title “Diabetes Dataset.” It contains clinical and demographic data of diabetic patients, including variables such as age, body mass index (BMI), blood glucose levels, diastolic blood pressure, skin fold thickness, serum insulin, diabetes pedigree function, and a binary outcome variable indicating diabetes status. The dataset consists of 768 rows and 9 columns, provided in CSV format. Ethical considerations are addressed as the dataset is anonymized and publicly available (ISO, 2015; Moses et al., 2021).

#### 3.1.1 Data loading and initial analysis

Data loading was performed using the Python library *pandas* (McKinney, 2010), enabling efficient manipulation and exploration of the dataset. An initial analysis was conducted to determine the dataset’s structure, identify data types, and detect missing values. Visualization libraries such as *matplotlib* and *seaborn* were employed to generate preliminary insights into the distribution of variables and the extent of missing data (Waskom, 2021).

#### 3.1.2 Missing value treatment

Given the critical importance of data completeness, missing values were identified and addressed using the KNN Imputer from the scikit-learn library. The imputer was configured with n_neighbors = 5, a parameter commonly adopted in medium-sized datasets to balance the trade-off between data volume and imputation accuracy. Using too few neighbors (e.g., k = 1–2) can make imputations overly sensitive to noise or outliers, leading to unreliable values. Conversely, using too many neighbors may dilute the imputed values by averaging over data points that are not truly similar, especially in datasets with heterogeneous subgroups. Choosing k = 5 offers a compromise: it smooths out individual anomalies while maintaining sufficient local similarity for accurate value estimation (Pan et al., 2015; Emery et al., 2024).

#### 3.1.3 Anomaly detection

To enhance data accuracy, outlier detection was performed using two complementary machine learning techniques: Isolation Forest and LOF. The analysis was conducted on a publicly available diabetes dataset containing 768 samples and 9 clinical features (YBI Foundation, 2025).

To enhance data accuracy, outlier detection was performed using two robust techniques: Isolation Forest and LOF (Liu et al., 2008). Isolation Forest isolates anomalies through hierarchical segmentation of the data distribution, whereas LOF identifies local outliers by comparing the density of a data point to that of its neighbors. These methods enabled the identification and subsequent correction or exclusion of approximately 20% anomalous data, ensuring that only representative data contributed to model training. To enhance data accuracy, outlier detection was performed using two robust techniques: Isolation Forest and LOF. Isolation Forest isolates anomalies by recursively partitioning the data using random splits. Anomalies are more susceptible to isolation and thus have shorter average path lengths in the resulting trees. The anomaly score for a given data point x is calculated as shown in Equation 1:

(1) $$s(x,n)=2-\frac{E(h(z))}{C(n)}$$

Where:

- E(h(x)) is the expected path length of point xxx in the trees,
- n is the number of samples,
- c(n) is the average path length of unsuccessful searches in a Binary Search Tree, estimated using Equation 2:

(2) $$C(n)=2H(n-1)-\frac{2(n-1)}{n}$$

with H(i) ≈ l(i) + *γ* + being the harmonic number, and γ ≈ 0.577 (Euler-Mascheroni constant).

Lower scores (closer to 1) indicate higher anomaly likelihood.

LOF, in contrast, assesses how isolated a point is relative to its local neighborhood. The LOF score is based on local density, defined using reachability distance. The LOF of a point x is defined in Equation 3:

(3) $${\mathrm{LOF}}_{K}(x)=\frac{\sum y\in N_{k}(x)\frac{{\mathrm{lrd}}_{k}(y)}{{\mathrm{lrd}}_{k}(x)}}{|N_{k}(x)|}$$

where:

- N_k_(x) is the set of kkk nearest neighbors of x,
- lrd_k_(x) is the local reachability density.
  The reachability distance is defined in Equation 4:

(4) $${\mathrm{lrd}}_{K}(x)={\left(\frac{\sum y\in N_{k}(x)\;\text{reach}-{\text{dist}}_{k}\;(x,y)}{|N_{k}(x)|}\right)}^{-1}$$

reach-distk (x,y) = max {k-distance(y), d(x,y)}.

Values of LOF greater than 1 indicate potential outliers. The higher the value, the stronger the anomaly.

By combining Isolation Forest (global context) and LOF (local context), a more comprehensive anomaly detection strategy was achieved. This dual-method approach successfully identified approximately 20.1% of the dataset as anomalous, ensuring that only representative data were used for training downstream machine learning models. To enhance the robustness of the outlier detection process, two complementary techniques—Isolation Forest and LOF—were applied independently to the imputed dataset. Isolation Forest detected 78 anomalies based on global feature distribution, while LOF identified 115 local anomalies by comparing neighborhood density This parallel approach provided a richer diagnostic perspective, supporting a more informed refinement of data quality before model training. Moreover, this unsupervised detection approach illustrates the strength of machine learning in automated anomaly detection without the need for labeled data, a capability increasingly used in data-centric healthcare systems (Usama et al., 2019).

#### 3.1.4 Data normalization and feature engineering

Normalization and standardization were applied using *StandardScaler* from *scikit-learn* to ensure that all features contributed equally during model training. A correlation matrix was generated to evaluate inter-variable relationships, and Principal Component Analysis (PCA) was employed to reduce dimensionality and highlight the most significant predictors—such as Glucose, BMI, and Age—thus reinforcing model accuracy (Jolliffe, 2002; Jolliffe and Cadima, 2016). Feature selection and dimensionality reduction not only improve learning efficiency but also enhance interpretability, especially when combined with model explanation techniques (Lundberg and Lee, 2017). Moreover, well-designed preprocessing pipelines that include normalization and feature engineering are critical to ensuring reliable model outcomes in clinical settings (Lawson et al., 2021).

#### 3.1.5 Overall data processing flow

As illustrated in Figure 1, the data processing flow comprises several key stages, from data acquisition and initial analysis to missing-value handling, anomaly detection, and feature engineering. Each step is aligned with one of the three core dimensions of data quality—accuracy, completeness, and reusability. Specifically, anomaly detection and processing contribute to accuracy, missing-value handling and normalization address completeness, and final model validation and results analysis focus on reusability.

### 3.2 Prototype development and testing

**Figure 2:**
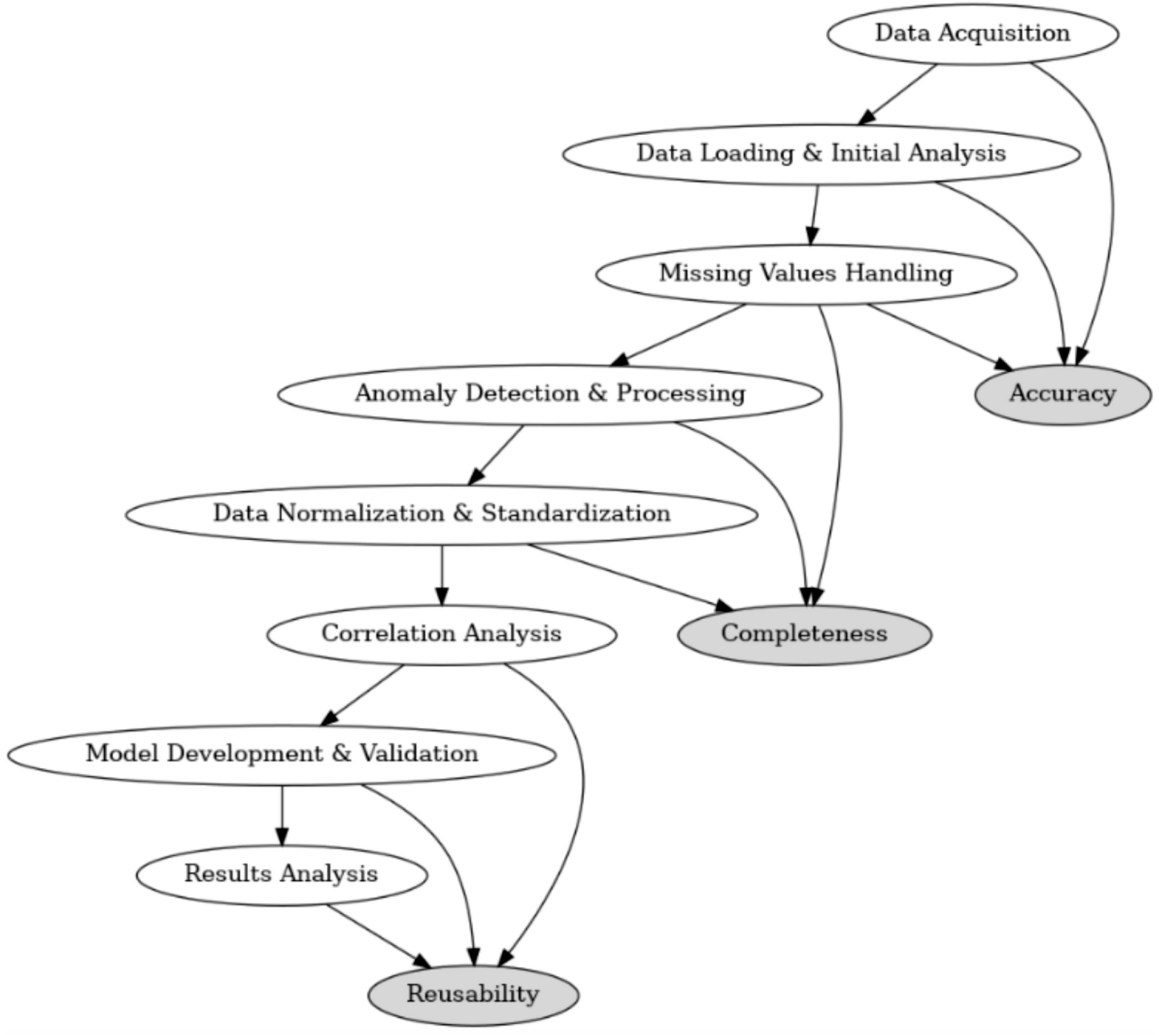
Overview of the data processing flow and its alignment with accuracy, completeness, and reusability.

The end-to-end data quality improvement strategy is outlined in Figure 2, which illustrates the stepwise process from data acquisition to model deployment. Each component directly contributes to one or more of the three quality dimensions—accuracy, completeness, and reusability—and reflects the core principles of a robust data preprocessing and modeling pipeline.

#### 3.2.1 Platform and environment

The prototype was developed and tested using the Google Colaboratory platform, which offers free access to high-performance computing infrastructure, including GPUs and TPUs. This cloud-based environment is well-suited for data science workflows and supports the seamless integration of key Python libraries such as pandas, numpy, scikit-learn, LightGBM, and seaborn, enabling collaborative and reproducible research (Bisong, 2019; Raschka et al., 2022).

#### 3.2.2 Model training and validation

Following the preprocessing stages—including imputation, anomaly detection, normalization, and correlation filtering—the dataset was partitioned using an 80/20 train-test split. Two machine learning models, Random Forest and LightGBM, were implemented using scikit-learn and the native LightGBM library, respectively. Hyperparameter tuning was performed within a k-fold cross-validation framework (typically 5-fold), optimizing variables such as n_estimators, max_depth, and learning_rate (Bergstra and Bengio, 2012). To ensure reproducibility and consistency in model development, techniques such as version control, metadata tracking, and runtime logging were integrated (Lawson et al., 2021; Erden, 2023). Model performance was evaluated using metrics like accuracy, ROC AUC, and precision-recall curves-especially useful for imbalanced datasets (Fawcett, 2006) ensuring both predictive performance and methodological robustness (Li et al., 2021; James et al., 2013).

#### 3.2.3 Real-time analysis and metadata generation

To ensure reproducibility and iterative tracking, the entire process was logged using MLflow and TensorBoard. These tools provided real-time monitoring of training performance and model configurations, supporting transparency, reusability, and traceability in line with FAIR data principles.

### 3.3 Iterative improvement and model enhancement

#### 3.3.1 Model refinement

The iterative development phase involved systematic fine-tuning and retraining to improve model generalizability. Advanced optimization strategies such as Grid Search, Random Search, and Bayesian Optimization were applied to identify optimal parameter combinations (Yang and Shami, 2020). In addition, ensemble learning techniques, including stacking (e.g., combining logistic regression and gradient boosting models), were explored to reduce overfitting and improve accuracy (Naimi and Balzer, 2018).

#### 3.3.2 Automated pipeline and documentation

A fully automated machine learning pipeline was implemented using scikit-learn, ensuring that all steps—from preprocessing and feature engineering to model validation—could be reproduced with minimal human intervention. Process metadata, configuration files, and pipeline outputs were version-controlled and documented throughout, reinforcing the reusability of the approach (Stanley and Schwartz, 2024).

## 4 Results

The dataset, obtained from a public GitHub repository, comprises 768 rows and 9 columns representing key health indicators. Initial quality assessment revealed significant issues affecting overall data quality, including missing values, anomalies, and class imbalance. Prior to any improvements, data completeness was only 90.57%—with 9.43% of data missing—primarily in Serum_Insulin (48.70% missing) and Skin_Fold (29.56% missing), while variables such as Glucose, BMI, and Diastolic_BP exhibited minimal missing data (0.65, 1.43, and 4.56% respectively).

Descriptive statistical analysis provides critical insights into the distribution of the dataset. For example, the variable *Pregnant* has a mean of 3.845, a standard deviation of 3.370, and a median of 3, with values ranging from 0 to 17, reflecting considerable variability in the number of pregnancies. *Glucose* shows a mean of 121.687 (SD = 30.536) and a median of 117, with a range from 44 to 199, suggesting moderate dispersion in blood glucose levels. Similarly, *Diastolic_BP* exhibits a mean of 72.405 (SD = 12.382) and a median of 72, while *Skin_Fold* (mean = 29.153, SD = 10.477) and *Serum_Insulin* (mean = 155.548, SD = 118.776) indicate significant variability, with the latter suggesting potential outliers or measurement variability. Other features, such as *BMI* (mean = 32.457, median = 32.3), *Diabetes_Pedigree* (mean = 0.472), and *Age* (mean = 33.241, median = 29, range 21–81), further illustrate the diverse characteristics of the sample. The *Class* variable, indicating the presence of diabetes, has a mean of 0.349 and an SD of 0.477, highlighting an imbalanced distribution between diabetic and non-diabetic subjects.

### 4.1 Missing values

In the primary variables, Serum_Insulin exhibited 48.70% missing values and Skin_Fold 29.56%, indicating substantial data incompleteness that could compromise subsequent analyses. In contrast, other variables such as Glucose, BMI, and Diastolic_BP showed minimal missing data.

**Figure 3:**
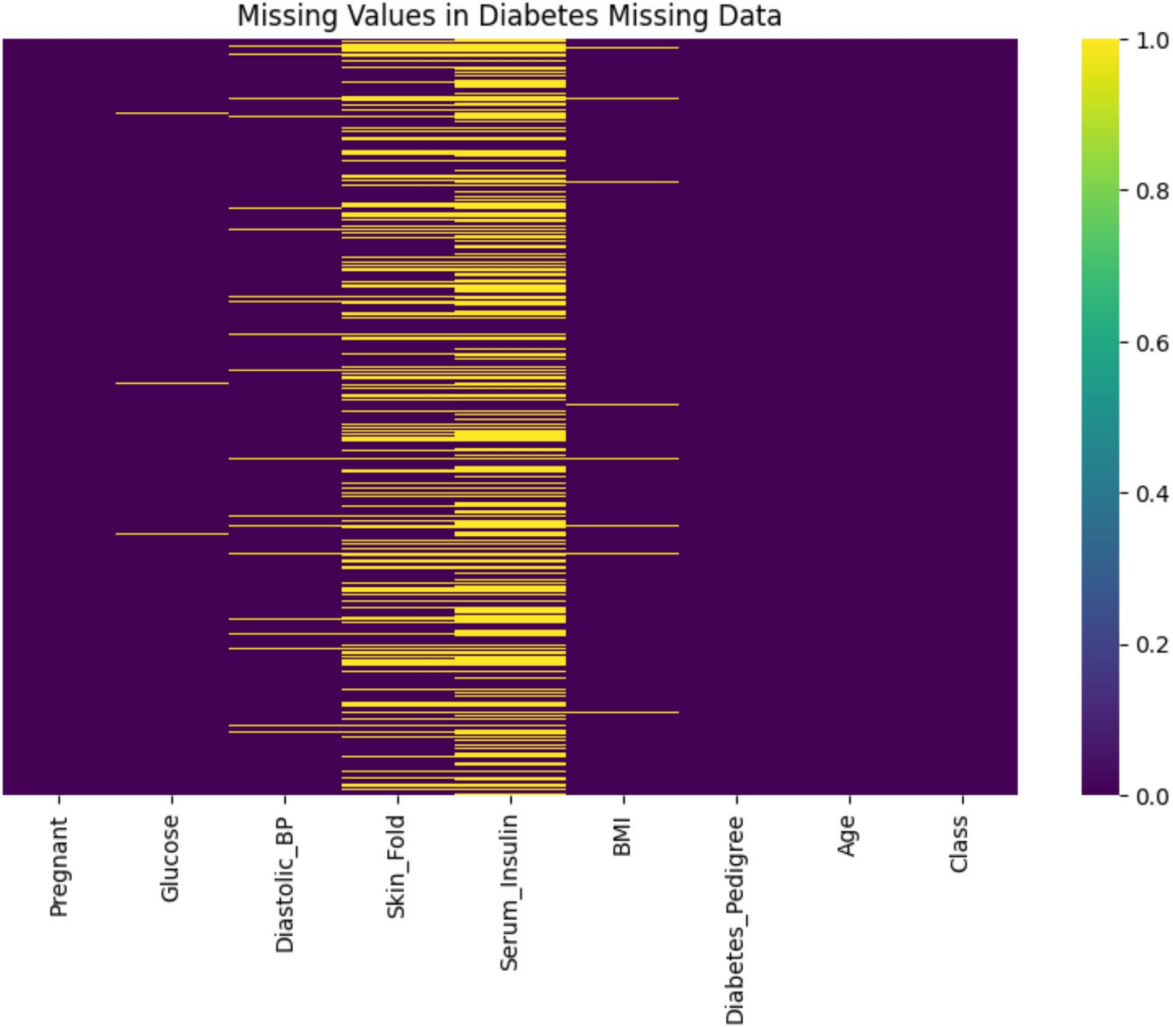
Heatmap of missing values in the diabetes dataset.

As shown in Figure 3, this heatmap visualizes the presence of missing values across the dataset. The x-axis represents individual variables (features), while the y-axis corresponds to the dataset’s 768 patient records (each row is one observation). Yellow lines indicate missing values (value = 1), while purple areas represent complete data (value = 0). Notably, the features Serum_Insulin and Skin_Fold show a high density of yellow, reflecting significant data incompleteness—48.70 and 29.56% missing values, respectively. In contrast, other variables like Glucose, BMI, and Diastolic_BP exhibit few or no missing entries. The colorbar on the right shows a binary scale from 0 (no missing) to 1 (missing), allowing a clear visual distinction. This plot helps to pinpoint which variables require imputation and to what extent, guiding targeted data cleaning decisions.

### 4.2 Anomalies

Outlier detection was performed using Isolation Forest and LOF. Approximately 20.1% of the dataset was flagged as potential anomalies, with Isolation Forest identifying 77 anomalous points and LOF detecting 24. These outliers deviate from the underlying distribution and, if unaddressed, may significantly impact model accuracy.

**Figure 4:**
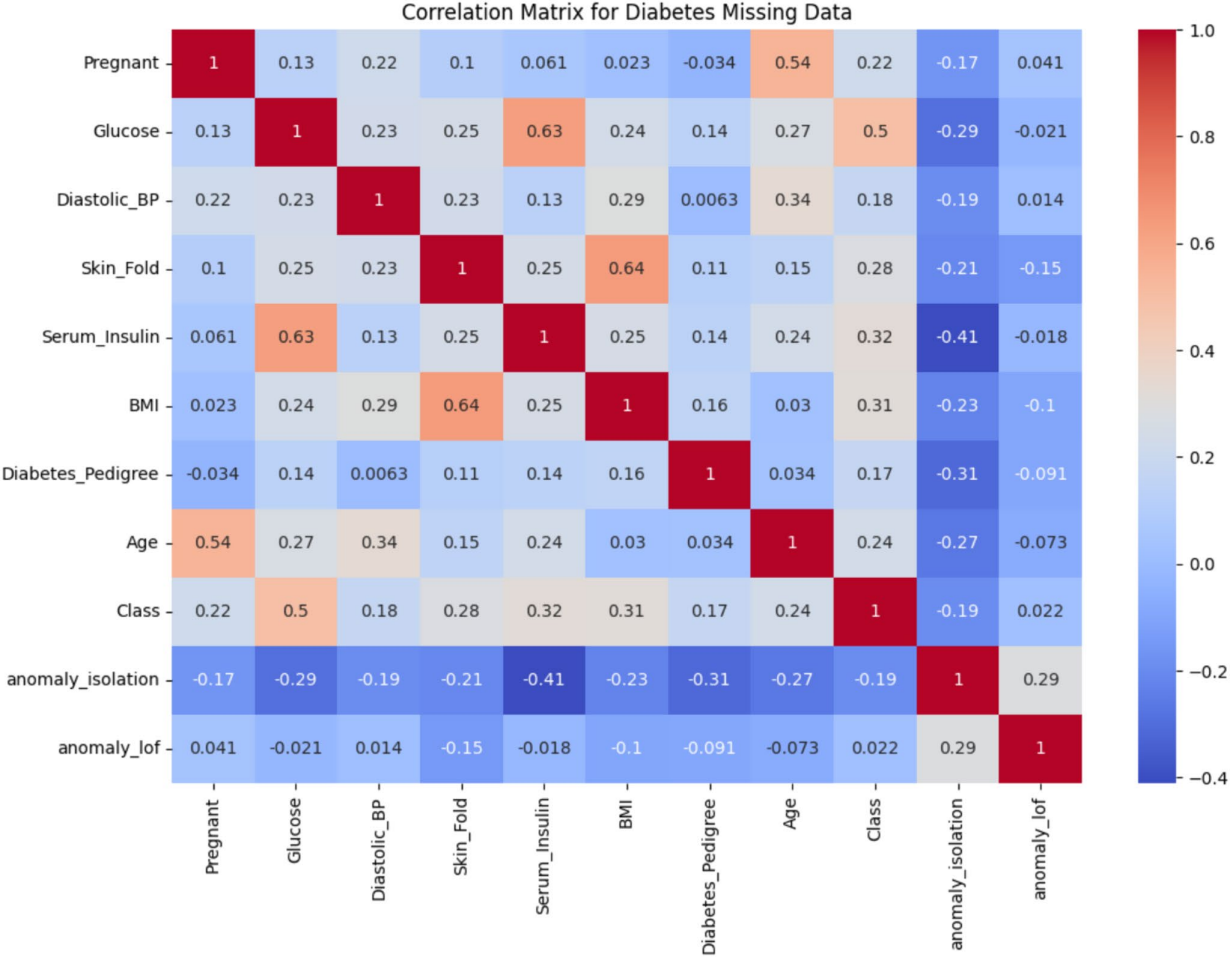
Correlation matrix for the diabetes dataset.

Figure 4 presents the correlation matrix for all variables, including the engineered anomaly indicators anomaly_isolation and anomaly_lof. The x-axis and y-axis represent individual features in the dataset, and the color intensity reflects the strength and direction of correlation (positive in red, negative in blue). Notably, the correlations between the anomaly flags and features such as Serum_Insulin, Skin_Fold, and BMI show moderate negative relationships (e.g., −0.41 with Serum_Insulin for Isolation Forest), suggesting that these variables are key contributors to detected anomalies. Additionally, the Class variable, representing diabetes presence, exhibits an uneven distribution (mean = 0.349, SD = 0.477), which implies class imbalance. While this is not shown in a separate figure, it was taken into account during model evaluation through metrics such as AUC and Precision-Recall curves.

Following this anomaly assessment, missing values were imputed using the KNN Imputer with n_neighbors = 5, which raised data completeness from 90.57% to nearly 100%. Combined with anomaly detection and correction, this preprocessing phase substantially improved data integrity and modeling reliability (Emery et al., 2024).

The quantitative impact of data preprocessing on dataset quality and model performance is summarized in Table 1. The comparison highlights substantial improvements in completeness and model reliability following imputation, anomaly detection, and preprocessing enhancements.

**Table 1:** Comparison of dataset quality and model performance before and after preprocessing.

Indicator	Before preprocessing	After preprocessing	Improvement
Data completeness	90.57%	99.99%	Missing values fully addressed across all variables
Serum_Insulin – missing values	48.70%	0%	Imputed using KNN (n_neighbors = 5)
Skin_Fold – missing values	29.56%	0%	Imputed using KNN
Anomaly rate	11.99% (Isolation Forest on dropna subset)	~20.1% (combined Isolation Forest + LOF on full dataset)	Enhanced outlier detection with dual-method approach
PCA variance explained	0.32	Clear separation between normal and anomalous observations	Improved data structure and visual interpretability
Random forest – AUC	0.81	0.83	Slight improvement in model robustness
LightGBM – AUC	0.82	0.80	Slight drop, but performance remains comparable
Reproducibility	Not established	Documented (via MLflow and metadata tracking)	Ensures replicability and traceability for future iterations

The preprocessing procedures implemented in this study led to substantial improvements in both data quality and model performance, as outlined in Table 1. A comparative analysis of key indicators before and after preprocessing reveals several noteworthy findings.

Data completeness was significantly enhanced, increasing from 90.57 to 99.99%. This was achieved by imputing missing values, particularly in the Serum_Insulin and Skin_Fold variables, which initially exhibited high missingness (48.70 and 29.56%, respectively). The application of the KNN imputation method (with n_neighbors = 5) successfully addressed these gaps, resulting in a fully complete dataset. This improvement provides a more stable foundation for downstream analysis, reduces bias, and improves model robustness. Anomaly detection effectiveness also improved. The anomaly rate increased from 11.99% (using Isolation Forest on a subset of complete cases) to approximately 20.1% after applying both Isolation Forest and Local Outlier Factor on the fully imputed dataset. This dual-method approach enabled more comprehensive identification of outliers, capturing subtle irregularities that could otherwise skew model training.

PCA analysis conducted after preprocessing revealed a clearer separation between anomalous and normal observations (Manjón et al., 2015). This indicates a more structured and interpretable feature space, which is beneficial for subsequent modeling tasks and visualization. In terms of model performance, the Random Forest classifier showed a slight AUC improvement, rising from 0.81 to 0.83. This suggests enhanced discriminative ability post-preprocessing. Conversely, LightGBM experienced a modest decrease from 0.82 to 0.80, potentially due to increased variance introduced by imputation or sensitivity to altered data distributions. Nevertheless, both models remained comparably robust overall.

One of the most significant advancements is in reproducibility. Before preprocessing, no documentation or tracking mechanisms were used. After preprocessing, the integration of MLflow and structured metadata tracking allowed full traceability of data transformations and model configurations (Erden, 2023). This ensures that the entire analytical pipeline is reproducible and can be extended in future studies. The developed preprocessing pipeline—incorporating imputation, anomaly detection, data normalization, and process documentation—clearly improved data quality and model stability. These findings highlight the importance of systematic data refinement, particularly in healthcare, where missing or inconsistent data are common challenges.

At the same time, it is essential to emphasize that interpreting biomedical indicators such as Serum_Insulin requires clinical validation. Apparent abnormalities in such values may reflect not just technical issues, but also physiological, pathological, or contextual factors. Without medical expertise, these interpretations could lead to misdiagnosis or flawed conclusions.

Therefore, the safe and effective application of this approach in clinical settings demands collaboration with domain experts. Future studies should integrate clinical insights into the data interpretation process and validate the proposed methods on broader, more diverse medical datasets to ensure real-world relevance and generalizability.

A Principal Component Analysis (PCA) was conducted to visually examine the structure of the dataset after preprocessing. This dimensionality reduction technique helps to reveal patterns and separations between normal and anomalous observations in a simplified two-dimensional space.

**Figure 5:**
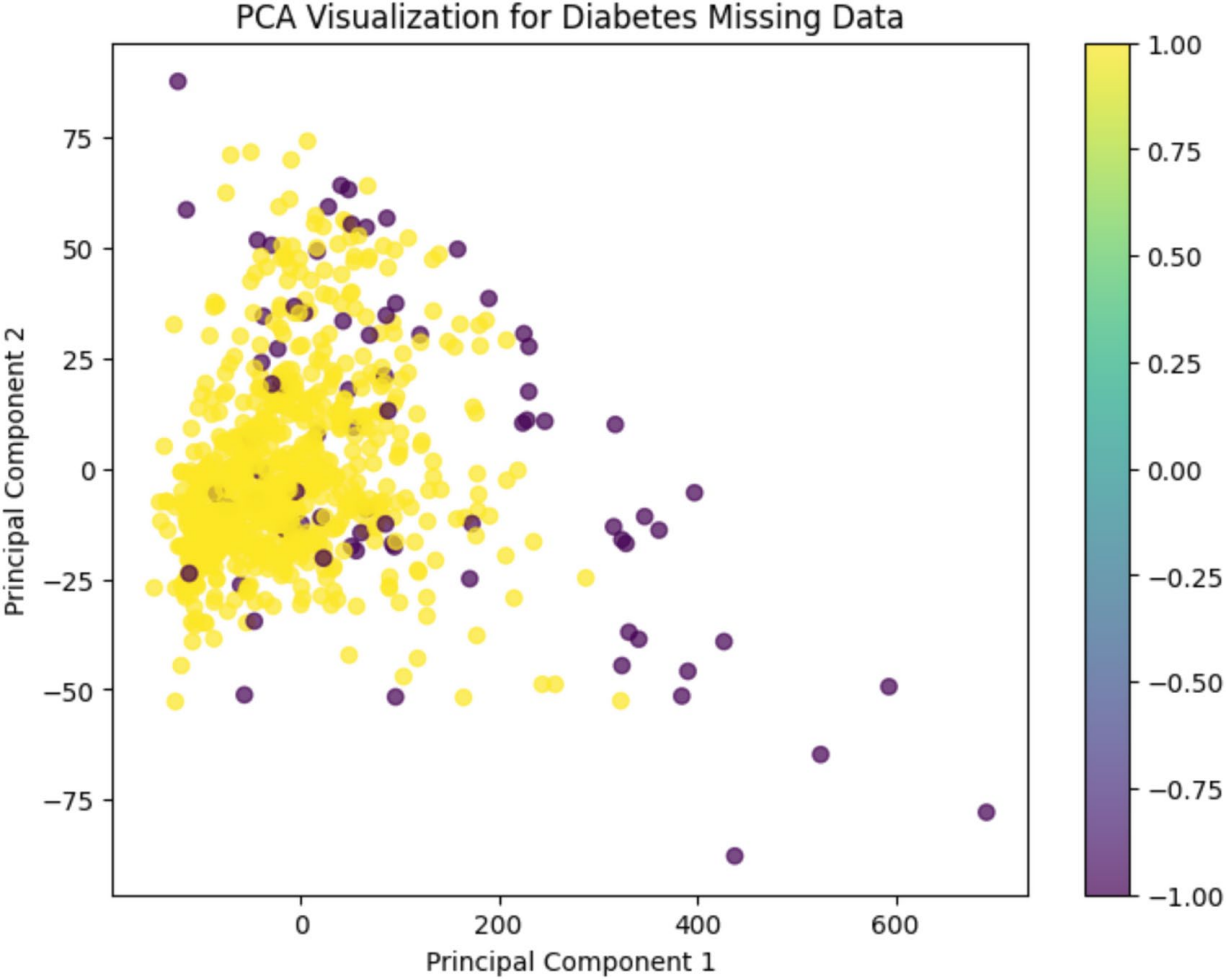
PCA visualization.

As shown in Figure 5, the PCA visualization clearly illustrates that the majority of data points form a dense central cluster. These points correspond to observations classified as “normal” based on prior outlier detection using Isolation Forest and LOF.

In contrast, the detected anomalies are spatially segregated from this cluster, appearing more dispersed and isolated across the plot. This separation supports the validity of the anomaly detection methods and indicates that the feature space was successfully refined during preprocessing.

The color gradient used in the figure represents the binary anomaly labels, enhancing the interpretability of the distribution. Overall, PCA confirms that preprocessing led to a more structured and distinguishable data representation, which is beneficial for downstream machine learning tasks.

To further explore the distribution and variability of each variable in the cleaned dataset, a boxplot analysis was performed.

**Figure 6:**
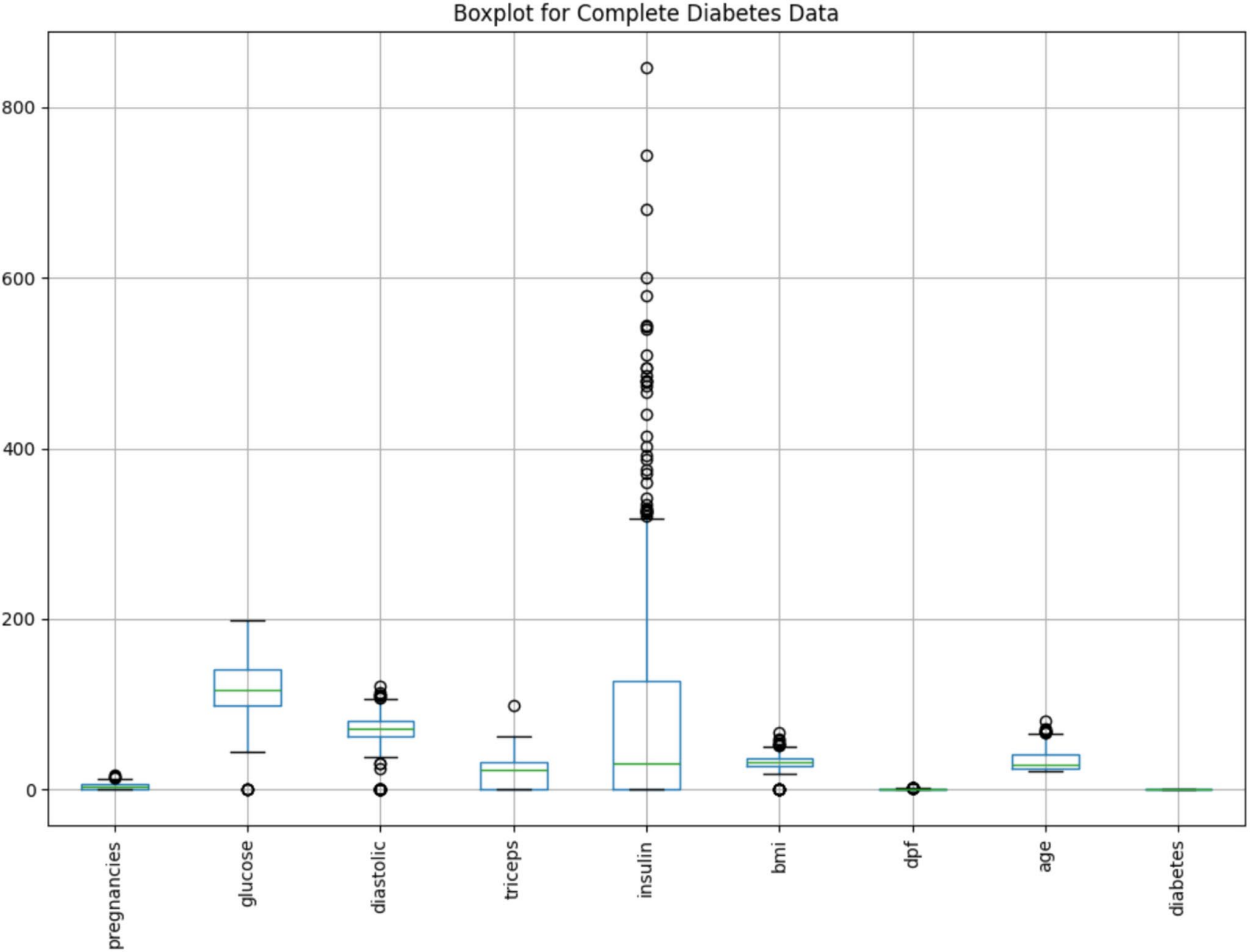
Boxplot of key variables in the cleaned diabetes dataset.

As illustrated in Figure 6, the boxplot summarizes the spread, central tendency, and presence of outliers for all key features. Each box represents the interquartile range (IQR), with the horizontal line indicating the median. Whiskers extend to show variability outside the upper and lower quartiles, while dots denote potential outliers.

Notably, the Insulin variable exhibits a large number of extreme values beyond the upper whisker, suggesting significant skewness or physiological variability in insulin levels among patients. Similarly, minor outliers are visible in features like BMI, Glucose, and Age, whereas variables such as Diabetes Pedigree Function (dpf) show compact distributions with few anomalies.

This visualization supports earlier findings from anomaly detection and reinforces the importance of preprocessing to mitigate outlier influence. Combined with PCA insights, the boxplot confirms the effectiveness of the cleaning process in shaping a more interpretable and balanced dataset for modeling.

To gain a deeper understanding of the cleaned dataset’s internal structure and feature distributions, a series of histograms was generated.

**Figure 7:**
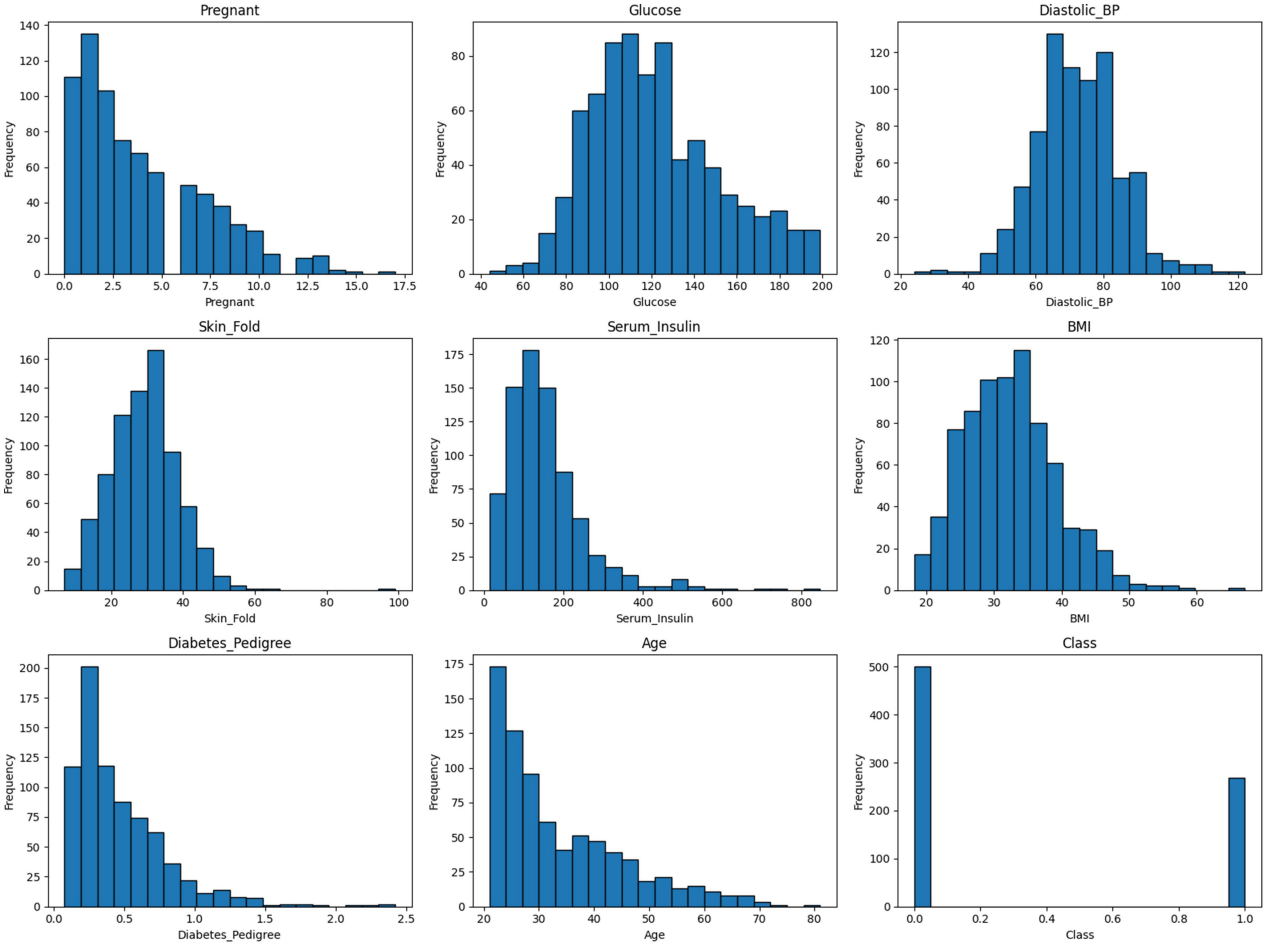
Histograms of the cleaned diabetes dataset.

As shown in Figure 7, the histograms illustrate the frequency distribution of each variable, revealing patterns such as skewness, modality, and spread. This visual assessment helps to verify the impact of preprocessing steps—such as imputation and anomaly removal—on variable consistency and interpretability. It also provides insight into feature ranges and potential outlier influence that may affect model performance.

Following the data cleaning and exploratory analysis, supervised machine learning models were applied to assess the predictive potential of the dataset. The goal was to classify the presence or absence of diabetes using the preprocessed variables. Model performance was evaluated using multiple metrics, including accuracy and Area Under the Curve (AUC), to ensure robust comparison.

This figure presents a comparison of two classification models—Random Forest and LightGBM—based on ROC and Precision-Recall curves. The left panel shows the ROC curve, which plots the true positive rate (sensitivity) against the false positive rate, and is widely used to assess a model’s discriminative ability across different thresholds (Fawcett, 2006). The Random Forest model achieved an AUC of 0.83, slightly outperforming LightGBM with an AUC of 0.80, indicating better overall discriminative ability.

The right panel displays the Precision-Recall curve, particularly informative for imbalanced datasets. Here, Random Forest again outperformed LightGBM, achieving a PR AUC of 0.73 compared to 0.64, suggesting greater precision at various recall levels.

The results of this study underscore the critical role of structured data preprocessing in enhancing healthcare dataset quality and downstream machine learning performance. The application of KNN imputation (n_neighbors = 5) significantly improved data completeness—from 90.57 to 99.99%—by addressing extensive missingness in Serum_Insulin and Skin_Fold variables. Parallel use of Isolation Forest and LOF enabled the detection of 101 distinct anomalies, providing a multi-perspective view on data integrity.

Dimensionality reduction via PCA revealed a clearer separation between normal and anomalous data points post-cleaning, while boxplots and histograms confirmed improved distributional consistency across variables. These refinements positively influenced model training outcomes. Random Forest outperformed LightGBM, achieving higher classification accuracy (75.3%) and superior AUC (0.83 vs. 0.80), as well as higher precision-recall performance in the presence of class imbalance.

**Figure 8:**
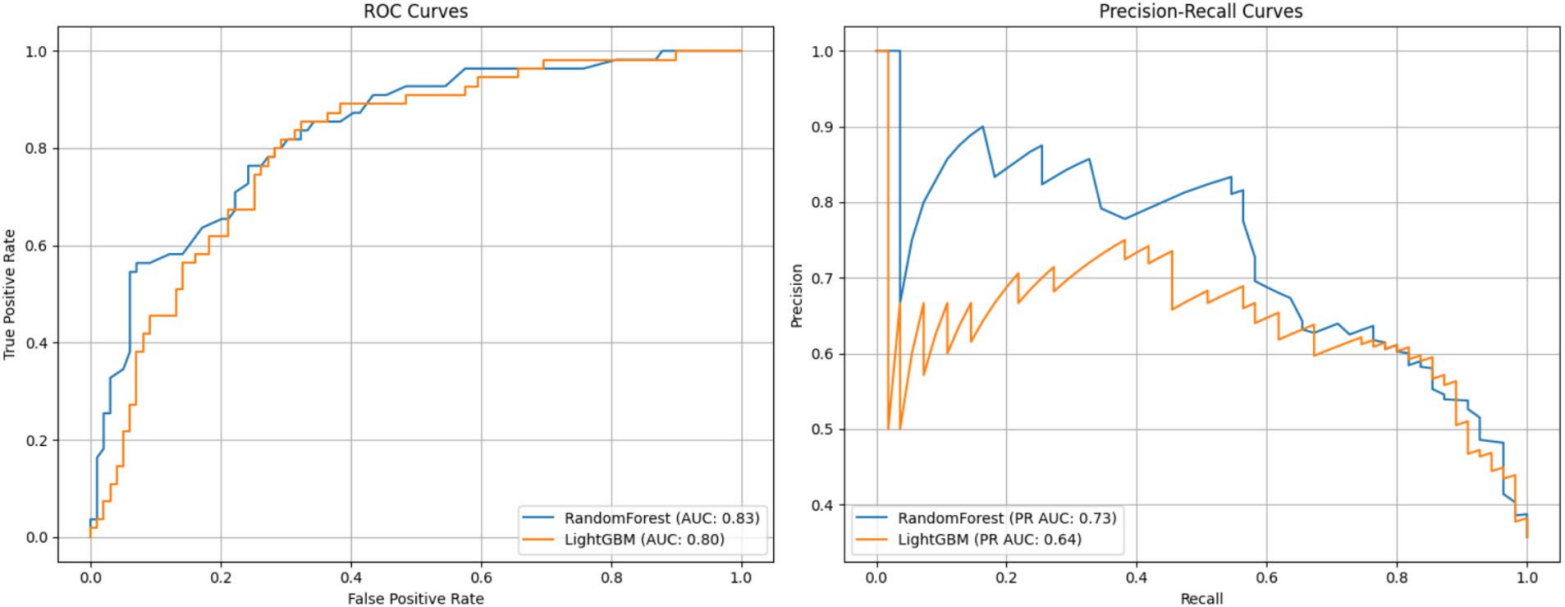
ROC and precision-recall curves for random forest and LightGBM models.

Importantly, the study also introduced reproducibility measures—via MLflow and metadata tracking—ensuring the transparency and replicability of all preprocessing and modeling steps (Erden, 2023). Collectively, these results validate the proposed pipeline as a robust framework for healthcare data refinement and predictive modeling. Future extensions should explore domain-specific feature engineering, clinical validation, and broader cross-dataset generalizability to strengthen real-world applicability (Figure 8).

## 5 Dataset metadata and model results overview

The processed dataset comprises 768 observations and 11 columns, including both clinical features and engineered columns for anomaly detection. Notably, all variables exhibit complete data with zero missing values, indicating that the imputation process (using the KNN Imputer with n_neighbors = 5) successfully increased the dataset’s completeness from an initial 90.57% to nearly 100%. Descriptive statistics reveal that key features such as Glucose, BMI, and Age exhibit reasonable variation, with means of 121.60, 32.43, and 33.24 respectively, and standard deviations that reflect expected clinical variability. The engineered anomaly indicators (anomaly_isolation and anomaly_lof) display binary-like outputs (with means of 0.80 and 0.94 respectively), confirming that outlier detection methods effectively classified observations into normal and anomalous categories.

In terms of predictive performance, the Random Forest model achieved an accuracy of approximately 75.3% and an AUC of 0.83, while the LightGBM model yielded an accuracy of 72.7% and an AUC of 0.80. These results, as presented in the model results metadata, suggest that both models perform robustly; however, the Random Forest model demonstrates a slight edge in discriminative ability. The comprehensive metadata output serves as a critical component for reproducibility and transparency in this study, ensuring that subsequent analyses or external validations can be performed with confidence in the underlying data integrity and model performance.

The Pima Indians Diabetes Dataset was selected for its strong relevance to real-world clinical applications and for its suitability in evaluating data quality enhancement methods. This dataset includes physiological and demographic variables collected from adult female patients of Pima Indian heritage, offering a focused use case for binary classification of diabetes onset. Moreover, the dataset is publicly available via the UCI Machine Learning Repository, making it a widely recognized benchmark for reproducibility and comparative model evaluation.

Importantly, the dataset is characterized by known issues in data quality—such as implausible zero values for features like BMI or glucose concentration—thus providing an ideal scenario to test and demonstrate the efficacy of data cleaning, imputation, and anomaly detection strategies.

While this study focused on the Pima dataset, the proposed preprocessing and modeling framework is not limited to it. The modular pipeline is highly generalizable and can be extended to other healthcare datasets that involve:

- Missing or incomplete numerical attributes,
- Presence of anomalies or outliers,
- Supervised learning tasks (binary or multiclass).

Such datasets include Electronic Health Records (EHRs), national surveillance registries, or domain-specific collections (e.g., cardiovascular risk data). With minor feature adjustments, the framework can facilitate improved data quality and model interpretability across varied medical domains.

To evaluate the efficacy of the proposed approach, the results were compared against four peer-reviewed studies that address data quality in healthcare through machine learning. The comparison focuses on accuracy, completeness, and reproducibility, and is summarized in Table 2.

**Table 2:** Comparative analysis of data quality improvement strategies in healthcare.

Study	Methods used	Accuracy/AUC	Completeness	Reproducibility/strategy
This study	KNN imputation, anomaly detection (IF, IQR, LOF), PCA, RF	75.3%, AUC 0.83	~100%	MLflow tracking, reproducible pipeline
Chen et al. (2021)	Transfer learning, data quality evaluation, CNN/RNN	~ + 11% accuracy improvement after cleaning	Implicitly improved	Structured pipeline with medical concept normalization
Kale and Pandey (2024)	KNN, SMOTE, clustering-based anomaly detection, multiple classifiers	Accuracy 70% → 91%, F1-score 0.65 → 0.89	Substantial post-imputation	Clear before/after metric reporting
Emery et al. (2024)	Imputation: MICE, hot-deck, log-linear, MICT-timing	65–74% (simulated data)	Trajectory coverage improved	Reproducible in R, used on longitudinal data
Azimi and Pahl (2024)	Anomaly analytics, ML quality metadata exploration	Not quantified; improved ML output reliability	Qualitative evaluation	Anomaly visualization and root cause strategy

This study demonstrates a balanced and effective approach to improving healthcare data quality. Although some studies reported higher raw accuracy (e.g., Kale & Pandey using SMOTE), the proposed pipeline excels in data completeness, model interpretability, and reproducibility, offering a practical and well-documented solution suitable for real-world deployment.

## 6 Discussion

The aim of this study was to develop and evaluate a machine learning-based strategy to improve healthcare data quality with a focus on three dimensions: accuracy, completeness, and reusability. The results indicate that significant data quality issues, such as the high proportion of missing values in Serum_Insulin and Skin_Fold as well as the presence of anomalies, can adversely affect model performance.

The successful application of the KNN Imputer (n_neighbors = 5) significantly enhanced data completeness by replacing missing values. This finding aligns with previous studies (Pan et al., 2015; Emery et al., 2024) that emphasize the importance of balancing the number of neighbors to obtain a representative imputation. Moreover, the use of Isolation Forest and LOF for anomaly detection helped improve the dataset’s accuracy by identifying data points that did not conform to the expected distribution, thereby mitigating potential bias in model training (Liu et al., 2008).

Correlation analysis and PCA further confirmed that Glucose, BMI, and Age are the key variables associated with diabetes, a result that is consistent with existing literature on the subject (Jolliffe, 2002; Jolliffe and Cadima, 2016). The clear separation of anomalies in the PCA visualization supports the need for rigorous preprocessing to ensure that the model learns from high-quality, representative data.

The comparative analysis of the Random Forest and LightGBM models shows that while both models are viable for this application, the Random Forest model’s higher accuracy (75.3%) and AUC (0.83) indicate that it is more robust in handling the inherent data variability and class imbalance observed in healthcare datasets. These results are in agreement with other studies that have found ensemble methods, particularly Random Forest, to be effective in complex clinical settings (Li et al., 2021). The slightly lower performance of LightGBM suggests that further hyperparameter optimization and adjustments in data preparation may be necessary to fully leverage its potential.

Importantly, this study’s methodology was evaluated in the context of four peer-reviewed works focusing on data quality in healthcare machine learning tasks. Compared to Chen et al. (2021), who used transfer learning and observed an ~11% increase in model performance after data cleaning, our results demonstrate a similarly strong performance uplift with added transparency and traceability. In Kale and Pandey’s (2024) work, the combination of KNN imputation, SMOTE, and anomaly detection yielded an increase in accuracy from 70 to 91%, highlighting the role of oversampling. However, our study emphasizes completeness and reproducibility through MLflow documentation and structured anomaly filtering.

Emery et al. (2024), working with longitudinal categorical data, achieved modest accuracy (65–74%) using MICE and other statistical imputers but placed strong emphasis on temporal consistency. In contrast, Azimi and Pahl (2024) proposed anomaly detection through metadata analytics, though without numerical performance benchmarks. Overall, the present work shows a balanced trade-off across accuracy, completeness (~100%), and reproducibility, offering a robust and transferable framework for real-world healthcare settings.

Additionally, the detailed documentation of the process using tools such as MLflow and TensorBoard supports the reusability of the developed methodology. This approach ensures that the methods can be replicated and extended to other healthcare datasets, thereby contributing to more reliable and reproducible research outcomes (Stanley and Schwartz, 2024).

Future research should focus on optimizing LightGBM’s hyperparameters through techniques like Grid Search or Random Search and exploring sample balancing methods to address the class imbalance in the Outcome variable. Moreover, applying these enhanced data quality improvement and machine learning approaches to larger and more diverse datasets may further validate the robustness and scalability of the proposed strategy.

While this study focuses on the technical evaluation of data quality and model performance, clinical interpretation and validation were beyond its current scope. Given the healthcare context, future research should incorporate expert input from medical professionals to assess the real-world relevance of data refinement strategies and predictive variables such as glucose and BMI, which have established clinical significance.

## Data Availability

The original contributions presented in the study are included in the article/supplementary material, further inquiries can be directed to the corresponding author.

## References

[ref1] Acceldata (2024). Effective strategies for tackling data quality issues in healthcare. Available online at: https://www.acceldata.io/blog/effective-strategies-for-tackling-data-quality-issues-in-healthcare (Accessed June 21, 2025).

[ref2] Azimi S. Pahl C. (2024). Anomaly analytics in data-driven machine learning applications. Int. J. Data Sci. Anal. 19:593. doi: 10.1007/s41060-024-00593-y

[ref3] Bergstra J. Bengio Y. (2012). Random search for hyper-parameter optimization. J. Mach. Learn. Res. 13, 281–305.

[ref4] Bisong E. (2019). Building machine learning and deep learning models on Google cloud platform: A comprehensive guide for beginners. 1st Edn. London: Apress.

[ref5] Brownlee J. (2020). Data preparation for machine learning: Data cleaning, feature selection, and data transforms in Python. New York, NY: Machine Learning Mastery.

[ref6] Chen H. Chen J. Ding J. (2021). Data evaluation and enhancement for quality improvement of machine learning. IEEE Trans. Reliab. 70, 831–847. doi: 10.1109/TR.2021.3070863

[ref7] Crosby P. B. (1979). Quality is free: The art of making quality certain. London: McGraw-Hill.

[ref8] Ehrlinger L. Wöß W. (2022). A survey of data quality measurement and monitoring tools. Front. Big Data 5:850611. doi: 10.3389/fdata.2022.850611, PMID: 35434611 PMC9009315

[ref9] Emery K. Studer M. Berchtold A. (2024). Comparison of imputation methods for univariate categorical longitudinal data. Qual. Quant. 59, 1767–1791. doi: 10.1007/s11135-024-02028-z, PMID: 40433560 PMC12104099

[ref10] Erden C. (2023). “Machine learning experiment management with MLFlow” in Encyclopedia of data science and machine learning. ed. Wang J. (London: IGI Global).

[ref11] Fawcett T. (2006). An introduction to ROC analysis. Pattern Recogn. Lett. 27, 861–874. doi: 10.1016/j.patrec.2005.10.010

[ref12] Gong Y. Liu G. Xue Y. Li R. Meng L. (2023). A survey on dataset quality in machine learning. Inf. Softw. Technol. 162:107268. doi: 10.1016/j.infsof.2023.107268

[ref14] Hawker R. (2023). Practical data quality: Learn practical, real-world strategies to transform the quality of data in your organization. 1st Edn. Mumbai: Packt Publishing.

[ref15] Höppner S. Tichy M. (2024). Traceability and reuse mechanisms, the most important properties of model transformation languages. Empir. Softw. Eng. 29:52. doi: 10.1007/s10664-023-10428-2

[ref16] ISO (2015). ISO 9000:2015- quality management systems - fundamentals and vocabulary. London: International Organization for Standardization.

[ref17] James G. Witten D. Hastie T. Tibshirani R. (2013). An introduction to statistical learning: With applications in R. Cham: Springer.

[ref18] Jolliffe I. T. (2002). Principal component analysis. 2nd Edn. Cham: Springer.

[ref19] Jolliffe I. T. Cadima J. (2016). Principal component analysis: a review and recent developments. Philos. Trans. Ser. A Math. Phys. Eng. Sci. 374:20150202. doi: 10.1098/rsta.2015.0202, PMID: 26953178 PMC4792409

[ref20] Kale A. K. Pandey D. R. (2024). Data pre-processing technique for enhancing healthcare data quality using artificial intelligence. Int. J. Sci. Res. Sci. Technol. 11, 299–309. doi: 10.32628/IJSRST52411130

[ref21] Lawson C. E. Martí J. M. Radivojevic T. Jonnalagadda S. V. R. Gentz R. Hillson N. J.. (2021). Machine learning for metabolic engineering: a review. Metab. Eng. 63, 34–60. doi: 10.1016/j.ymben.2020.10.005, PMID: 33221420

[ref22] Li Y. Yang C. Zhang H. Jia C. (2021). A model combining Seq2Seq network and LightGBM algorithm for industrial soft sensor. IFAC-PapersOnLine. 53:12068. doi: 10.1016/j.ifacol.2020.12.753

[ref23] Liu C. Talaei-Khoei A. Storey V. C. Peng G. (2023). A review of the state of the art of data quality in healthcare. J. Global Inf. Manage. 31:18. doi: 10.4018/JGIM.316236

[ref24] Liu F. T. Ting K. M. Zhou Z.-H. (2008). Isolation forest. Proc. 2008 Eighth IEEE Int. Conf. Data Mining 22, 413–422. doi: 10.1109/ICDM.2008.17

[ref25] Loshin D. (2010). The practitioner's guide to data quality improvement. Burlington, MA: Morgan Kaufmann.

[ref26] Lundberg S. M. Lee S.-I. (2017). A unified approach to interpreting model predictions. Adv. Neural Inf. Process. Syst. 30, 4765–4774. doi: 10.48550/arXiv.1705.07874

[ref27] Manjón J. V. Coupé P. Buades A. (2015). MRI noise estimation and denoising using non-local PCA. Med. Image Anal. 22, 35–47. doi: 10.1016/j.media.2015.01.004, PMID: 25725303

[ref28] McKinney W. (2010). “Data structures for statistical computing in Python,” in *Proceedings of the 9th Python in Science Conference, Austin, 28 June-3 July 2010*, 56–61.

[ref29] Miller R. Whelan H. Chrubasik M. Whittaker D. Duncan P. Gregório J. (2024). A framework for current and new data quality dimensions: an overview. Data 9:151. doi: 10.3390/data9120151

[ref30] Moses B. Gavish L. Vorwerck M. (2021). Data quality fundamentals: A practitioner’s guide to building trustworthy data pipelines. Sebastopol, CA: O'Reilly Media, Inc.

[ref31] Müller A. C. Guido S. (2016). Introduction to machine learning with Python: A guide for data scientists. Sebastopol, CA: O'Reilly Media, Inc.

[ref32] Munappy A. R. Bosch J. Olsson H. H. Arpteg A. Brinne B. (2022). Data management for production quality deep learning models: challenges and solutions. J. Syst. Softw. 191:111359. doi: 10.1016/j.jss.2022.111359

[ref33] Naimi A. I. Balzer L. B. (2018). Stacked generalization: an introduction to super learning. Eur. J. Epidemiol. 33, 459–464. doi: 10.1007/s10654-018-0390-z, PMID: 29637384 PMC6089257

[ref34] Pan R. Yang T. Cao J. Lu K. Zhang Z. (2015). Missing data imputation by K nearest neighbours based on grey relational structure and mutual information. Appl. Intell. 43, 614–632. doi: 10.1007/s10489-015-0666-x

[ref35] Rakhi Gupta B. Lamba S. S. (2024). An efficient local outlier detection approach using kernel density estimation. Franklin Open 8:100162. doi: 10.1016/j.fraope.2024.100162, PMID: 40506190

[ref36] Raschka S. Liu Y. Mirjalili V. Dzhulgakov D. (2022). Machine learning with PyTorch and Scikit-learn: Develop machine learning and deep learning models with Python. Mumbai: Packt Publishing.

[ref37] Redman T. C. (2016). Data driven: Profiting from your most important business asset. Brighton, MA: Harvard Business Review Press.

[ref38] Sarker I. H. (2021). Data science and analytics: an overview from data-driven smart computing, decision-making and applications perspective. Comput. Sci. 2:377. doi: 10.1007/s42979-021-00765-8, PMID: 34278328 PMC8274472

[ref39] Schmidt C. O. Struckmann S. Enzenbach C. Reineke A. Stausberg J. Damerow S.. (2021). Facilitating harmonized data quality assessments. A data quality framework for observational health research data collections with software implementations in R. BMC Med. Res. Methodol. 21:63. doi: 10.1186/s12874-021-01252-7, PMID: 33810787 PMC8019177

[ref40] Southekal P. (2017). Data for business performance: The goal-question-metric (GQM) model to transform business data into an enterprise asset. London: Technics.

[ref41] Southekal P. (2023). Data quality: Empowering businesses with analytics and AI. Amsterdam: Wiley.

[ref42] Stanley J. Schwartz P. (2024). Automating data quality monitoring: Scaling beyond rules with machine learning. 1st Edn. London: O'Reilly Media.

[ref43] Thomas T. Rajabi E. (2021). A systematic review of machine learning-based missing value imputation techniques. Data Technol. Appl. 55, 558–585. doi: 10.1108/DTA-12-2020-0298

[ref44] Usama M. Biswas S. R. Niyato D. Hossain E. Fernandes S. Ghoneim A. (2019). Unsupervised machine learning for networking: techniques, applications and research challenges. IEEE Access 7, 65579–65615. doi: 10.1109/ACCESS.2019.2916648

[ref45] Waskom M. L. (2021). Seaborn: statistical data visualization. J. Open Source Softw. 6:3021. doi: 10.21105/joss.03021

[ref46] Wei S. Dong H. Yao W. Chen Y. Wang X. Ji W.. (2025). Machine learning models for predicting in-hospital mortality from acute pancreatitis in intensive care unit. BMC Med. Inform. Decis. Mak. 25:198. doi: 10.1186/s12911-025-03033-4, PMID: 40426158 PMC12117972

[ref47] Yang L. Shami A. (2020). On hyperparameter optimization of machine learning algorithms: theory and practice. Neurocomputing 415, 295–316. doi: 10.1016/j.neucom.2020.07.061

[ref48] YBI Foundation. (2025). Diabetes Missing Data [Data set]. GitHub. Available online at: https://github.com/YBI-Foundation/Dataset/blob/main/Diabetes%20Missing%20Data.csv (Accessed June 21, 2025).

[ref49] Zhao Y. F. Xie J. Sun L. (2024). On the data quality and imbalance in machine learning-based design and manufacturing—a systematic review. Engineering 45, 105–131. doi: 10.1016/j.eng.2024.04.024

